# Rhinitis induced to mace

**DOI:** 10.1186/2045-7022-1-S1-P11

**Published:** 2011-08-12

**Authors:** Gema Vanesa Sanchez Moreno, Soledad Terrados Cepeda, M Pilar Berges Gimenor, Maria Peña Peloche, Ricardo Madrigal Burgaleta, F Javier Sola Martinez, Dario Antolin Amerigo, Cristina Vlaicu Petruta, Emilio Alvarez-Cuesta

**Affiliations:** 1 Ramon y Cajal Hospital, Allergy Division, Madrid, Spain; 2 Ramon y Cajal Hospital, Head Allergy Division, Madrid, Spain

## Background

Spice allergy represents approximately 2 % cases of food allergy. There are many botanical families, but Apiaceae and Liliaceae had been commonly involved in cases of occupational asthma and food reactions in published literature.

## Methods

We present a case of 15-year-old male with peach allergy since childhood and grass pollen rhinitis, who showed cough and sneeze after eating spicy sausages on several times. These contained mace, ginger and garlic. After signing informed consent, oral controlled challenge with the same spicy sausages was positive so we developed allergy study.

## Results

Skin prick test to mace and ginger (10%) were positive and specific IgE to Pru p3 and mace were detected. Double-blind, placebocontrolled, oral food challenge (DBPCFC) with this spice showed rhinitis in few minutes and was negative to ginger. Through electrophoresis of peach, Pru p3, mace and ginger extracts were identified and the corresponding proteins purified and characterized as allergens. Mace extract was recognized by both anti-Pru p3 and antiTLPs antibodies, however immunoblotting study with patient sample demostrated proteins bands immunodetection in this extract with similar molecular weight to peach thaumatins. Finally skin prick tests with these purified allergens (Pru p 2.1 y 2.2) were positive.

## Conclusion

Thaumatin-like proteins are a new family of pollen and fruit allergens. Because of the sequence homologies between PR-5 proteins and thaumatin, an intensely sweet tasting protein isolated from the fruits of the West African rain forest shrub Thaumatococcus daniellii, these molecules are referred to as thaumatin-like proteins (TLP's). To our knowledge, this is the first report on the implication of TLPÂ´s in spice allergy. Unfortunately, information on fundamental aspects of the TLP family remains quite limited, this means that challenging fundamental and applied studies need to be conducted to characterize TLP's significance and clinical involvement.

